# RBM11 drives malignant progression of bladder cancer by regulating GNPDA1-PKM2 axis

**DOI:** 10.1016/j.isci.2026.115402

**Published:** 2026-03-17

**Authors:** Hang Tong, Tinghao Li, Junlong Zhu, Qian Dou, Qiong Yu, Yan Sun, Weiyang He

**Affiliations:** 1 Department of Urology, The First Affiliated Hospital of Chongqing Medical University, Chongqing 400016, China; 2 Department of Nephrology, The First Affiliated Hospital of Chongqing Medical University, Chongqing 400016, China; 3 Department of Anesthesiology, The First Affiliated Hospital of Chongqing Medical University, Chongqing 400016, China

**Keywords:** molecular physiology, cell biology, cancer

## Abstract

This study investigated the biological functions and molecular mechanisms of RNA-binding motif protein 11 (RBM11) in bladder cancer (BCa) progression. Integrated bioinformatics analysis of the TCGA database and validation in clinical tissues revealed that RBM11 is significantly upregulated in BCa and positively correlated with advanced tumor stage, poor prognosis, and epithelial-mesenchymal transition (EMT). RBM11 knockdown effectively suppressed migration, invasion, proliferation, and chemoresistance of BCa cells, whereas RBM11 overexpression produced opposite effects. Mechanistically, RBM11 promotes GNPDA1 expression by regulating alternative splicing of GNPDA. Furthermore, GNPDA1 directly interacts with PKM2 and inhibits its ubiquitin-proteasome-mediated degradation, thereby stabilizing PKM2 protein levels, enhancing glycolysis, and promoting malignant progression of BCa. Collectively, these findings indicate that RBM11 drives malignant progression of BCa through the GNPDA1-PKM2 axis, enhancing glucose metabolism reprogramming and EMT process, suggesting that RBM11 may be a potential therapeutic target for BCa.

## Highlights

• RBM11 promotes the proliferation, invasion, and metastasis of BCa
• RBM11 regulates GNPDA alternative splicing to stabilize PKM2 and promote glycolysis in BCa
• Inhibiting RBM11 can suppress the malignant progression of BCa

## Graphical abstract

**Figure:**
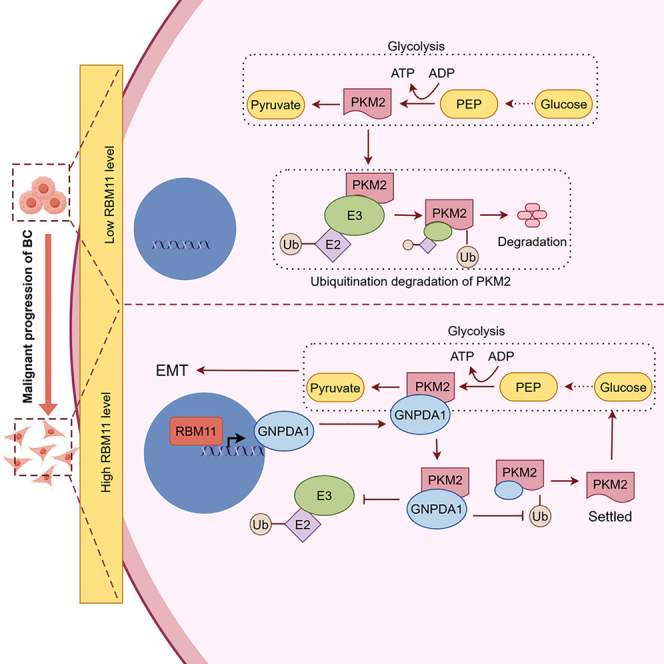

## Introduction

Bladder cancer (BCa) is a common malignancy of the urinary system.^1^ A substantial proportion of patients’ progress to muscle-invasive or advanced diseases, often accompanied by the development of chemotherapy resistance, resulting in poor prognosis and limited therapeutic options.^2^^,^^3^ Currently, extensive explorations have been conducted on the mechanisms underlying BCa progression, and evidence indicates that chimeric RNAs and post-transcriptional regulatory mechanisms play crucial roles in this process.^4^ BCa progression is also associated with dysregulation of multiple intracellular signaling pathways.^5^ In addition, recent studies increasingly recognize that metabolic reprogramming^6^ and tumor microenvironment (TME) remodeling^7^ are central drivers of BCa progression. However, the molecular mechanisms driving BCa progression remain incompletely understood, highlighting the urgent need to unravel key molecular pathways underlying disease progression for the development of effective therapeutic strategies.

Epithelial-mesenchymal transition (EMT) is a phenomenon of epithelial cells transforming into mesenchymal cells, which is a process of cell dedifferentiation or redifferentiation.^8^^,^^9^ EMT is a key molecular process that drives tumor invasion, metastasis, and treatment resistance.^10^ During EMT, cancer cells lose cell-cell adhesion and acquire migratory and invasive phenotypes, which are closely related to the invasive disease and adverse outcomes of BCa.^11^^,^^12^ The molecular basis of EMT in BCa is complex, involving multiple levels of dysregulation. Therefore, an in-depth study of the key molecules that drive EMT and enhance tumor cell migration, invasion, and chemoresistance in malignant BCa can provide insights for developing effective therapies to mitigate these issues and provide important targets for treating BCa.

As a key regulatory factor in RNA splicing, RBM11 plays an important role in post-transcriptional regulation.^13^ Recent studies have shown that RBM11 may participate in a variety of pathophysiological processes by regulating alternative splicing,^14^^,^^15^ but its biological function in tumorigenesis and development, especially in BCa, remains to be further explored. Preliminary analysis based on TCGA showed that the expression level of RBM11 in BCa tissues was significantly higher than that in normal adjacent tissues, and its expression level was closely related to tumor stage, grading, and patient prognosis. Notably, functional enrichment analysis further indicated that RBM11 may promote BCa progression by modulating the EMT process and metabolic reprogramming. Metabolic reprogramming, a hallmark of cancer, is characterized by enhanced aerobic glycolysis, which provides essential biosynthetic precursors and energy to support rapid proliferation of cancer cells.^16^^,^^17^ Concurrently, the EMT process enhances cellular invasion and migration capabilities, thereby driving malignant progression. Therefore, elucidating the precise molecular mechanisms by which RBM11 regulates EMT and metabolic reprogramming in BCa is of paramount importance for understanding disease pathogenesis and developing effective therapeutic strategies.

In this study, we show that RBM11 is significantly upregulated in BCa, and its expression level is positively correlated with the malignant progression of tumor cells. Importantly, RBM11 promotes the generation of its GNPDA1 subtype by regulating alternative splicing of the GNPDA gene. Upregulated GNPDA1 can directly bind to PKM2, inhibit its degradation via the ubiquitin-proteasome pathway, thereby enhancing PKM2 stability and activating glycolytic metabolic pathways. Collectively, our work elucidates a regulatory mechanism through which RBM11 drives BCa malignancy by orchestrating the GNPDA1-PKM2 axis.

## Results

### RBM11 is closely associated with the progression of BCa

**Figure 1:**
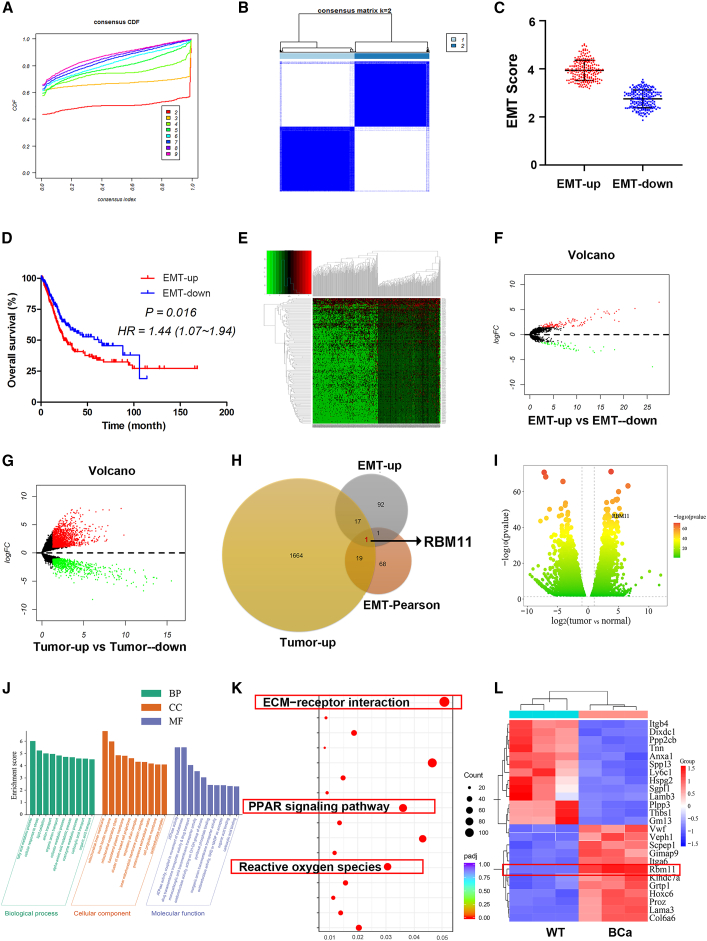
RBM11 is closely associated with the progression of BCa (A and B) Division of 405 BCa patients into EMT-up and EMT-down groups using the ConsensusCluster assay. (C and D) EMT scores and the OS periods of EMT-up and EMT-down groups (Kaplan-Meier method, *p* = 0.016). (E and F) TCGA data used to screen for DEGs between the EMT-up and EMT-down groups (fold change ≥ 2, FDR < 0.01). (G) TCGA data used to screen for DEGs between BCa tissues and normal bladder tissues (fold change ≥ 2, FDR < 0.01). (H) The intersection of the above gene clusters was analyzed, and RBM11 was ultimately identified. (I–L) Further validation using TCGA paired cancerous/paracancerous transcriptomic data confirmed high RBM11 expression in BCa tissues and significant enrichment in EMT-related pathways.

Based on the expression profiles of 200 EMT-associated genes, 405 BCa patients were stratified into EMT-up and EMT-down groups (Figures 1A–1C). Survival analysis revealed that patients in the EMT-down group had a significantly longer overall survival (OS) than those in the EMT-up group (Figure 1D). Using the TCGA database, we identified genes significantly upregulated in the EMT-up subgroup and those generally upregulated in tumor (Figures 1E–1G). A Biovenn analysis was then performed to intersect these gene sets with the predefined EMT-associated gene signature, which identified RBM11 as the sole overlapping gene (Figure 1H). Further validation using TCGA transcriptomic data from paired cancerous and paracancerous tissues confirmed that RBM11 was highly expressed in BCa tissues and significantly enriched in EMT-related pathways (Figures 1I–1L). These results strongly suggest that RBM11 is robustly associated with EMT in BCa.

### RBM11 is highly expressed in BCa and associated with advanced tumor stages

**Figure 2:**
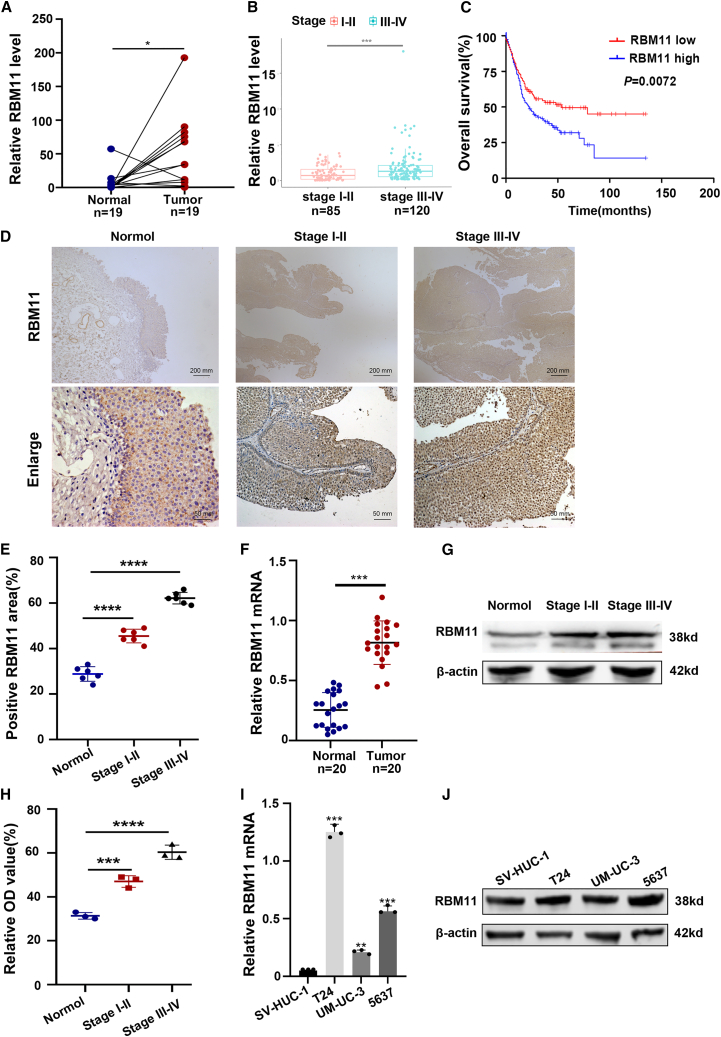
RBM11 is highly expressed in BCa and associated with advanced tumor stages (A) TCGA data used to screen for RBM11 between BCa tissues (*n* = 19) and normal bladder tissues (*n* = 19). Data are presented as mean ± SD, ∗*p* < 0.05 (paired *t* test). (B) Relationship between RBM11 and staging of BCa in TCGA data (stage I–Il *n* = 85; stage Ⅲ–IV *n* = 120). Data are presented as mean ± SD, ∗∗∗*p* < 0.001 (unpaired *t* test). (C) Relationship between RBM11 and OS in TCGA data (*n* = 405) (Kaplan-Meier method, *p* = 0.0072). (D and E) IHC of RBM11 in normal, stage I–Il and stage Ⅲ–IV groups (*n* = 3 technical replicates from 6 biological replicates for each group). Quantitative evaluation of RBM11 expression represented as area percentage. Scale bars: upper image, 200 μm; lower image, 50 μm. Data are presented as mean ± SD, ∗∗∗∗*p* < 0.0001 (one-way ANOVA). (F) RT-qPCR detection of RBM11 in different clinical samples groups (*n* = 3 technical replicates from 20 biological replicates for each group). Data are presented as mean ± SD, ∗∗∗*p* < 0.001 (paired *t* test). (G and H) WB detection of RBM11 in normal, stage I–Il, and stage Ⅲ–IV groups (*n* = 3 technical replicates from 3 biological replicates for each group). Data are presented as mean ± SD, ∗∗∗*p* < 0.001, ∗∗∗∗*p* < 0.0001 (unpaired *t* test). (I) RT-qPCR detection of RBM11 content in different cell lines (*n* = 3 technical replicates from three biological replicates for each group). Data are presented as mean ± SD, ∗∗*p* < 0.01, ∗∗∗*p* < 0.001 (unpaired *t* test). (J) WB detection of RBM11 content in SV-HUC-1, T24, UM-UC-3, and 5637 cell lines.

Analysis of the TCGA dataset demonstrated that RBM11 was significantly upregulated in tumor tissues and that its elevated expression was associated with advanced tumor stage and poorer OS (Figures 2A–2C). This stage-dependent upregulation was validated at the tissue level by IHC, which revealed intense nuclear staining in advanced tumors (Figures 2D and 2E), and confirmed by significantly higher mRNA and protein levels in stage III–IV samples (Figures 2F–2H). Consistent with clinical findings, BCa cell lines (T24, 5637, and UM-UC-3) exhibited higher RBM11 expression than the normal SV-HUC-1 cell line (Figures 2I and 2J). These findings strongly implicate RBM11 in BCa progression.

### RBM11 promotes migration, invasion, and chemoresistance in BCa cells

**Figure 3:**
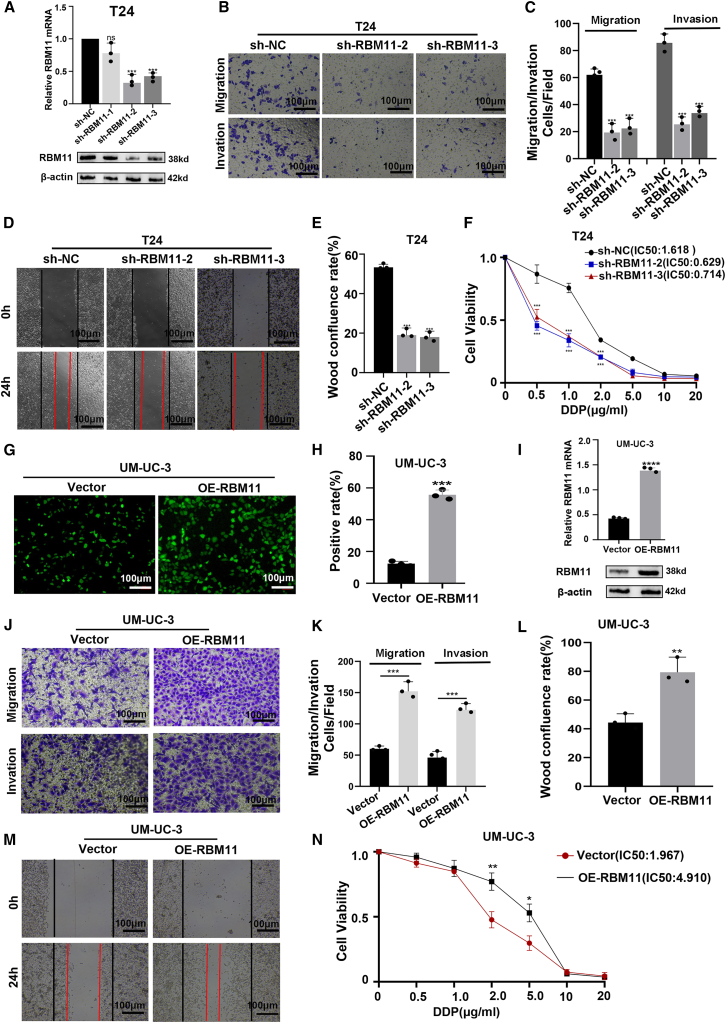
RBM11 promotes migration, invasion, and chemoresistance in BCa cells (A) RT-qPCR and WB detection efficiency of knocking down RBM11 (*n* = 3 technical replicates from three biological replicates for each group). Data are presented as mean ± SD, ∗∗∗*p* < 0.001 (one-way ANOVA). (B and C) Transwell assays demonstrated that knocking down RBM11 inhibits T24 cell migration and invasion (*n* = 3 technical replicates from three biological replicates for each group). Scale bars, 100 μm. Data are presented as mean ± SD, ∗∗∗*p* < 0.001 (one-way ANOVA). (D and E) Wound healing assay demonstrated that knocking down RBM11 inhibits T24 cell migration (*n* = 3 technical replicates from three biological replicates for each group). Scale bars, 100 μm. Data are presented as mean ± SD, ∗∗∗*p* < 0.001 (one-way ANOVA). (F) CCK8 assays demonstrated that knocking down RBM11 enhances T24 cell chemotherapy sensitivity (*n* = 3 technical replicates from 3 biological replicates for each group). Data are presented as mean ± SD, ∗∗∗*p* < 0.001 (one-way ANOVA). (G and H) Validation of RBM11 overexpression efficiency in UM-UC-3 cell line (*n* = 3 technical replicates from three biological replicates for each group). Scale bars, 100 μm. Data are presented as mean ± SD, ∗∗∗*p* < 0.001 (unpaired *t* test). (I) RT-qPCR and WB detection efficiency of RBM11 overexpression (*n* = 3 technical replicates from three biological replicates for each group). Data are presented as mean ± SD, ∗∗∗∗*p* < 0.0001 (unpaired *t* test). (J and K) Transwell assays demonstrated that RBM11 overexpression enhances UM-UC-3 cell migration and invasion (*n* = 3 technical replicates from three biological replicates for each group). Scale bars, 100 μm. Data are presented as mean ± SD, ∗∗∗*p* < 0.001 (unpaired *t* test). (L and M) Wound healing assay demonstrated that RBM11 overexpression enhances UM-UC-3 cell migration (*n* = 3 technical replicates from three biological replicates for each group). Scale bars, 100 μm. Data are presented as mean ± SD, ∗∗*p* < 0.01 (unpaired *t* test). (N) CCK8 assays demonstrated that RBM11 overexpression inhibits UM-UC-3 cell chemotherapy sensitivity (*n* = 3 technical replicates from three biological replicates for each group). Data are presented as mean ± SD, ∗*p* < 0.05, ∗∗*p* < 0.01 (unpaired *t* test).

To explore the function of RBM11 in BCa cells, we conducted knockdown and overexpression experiments in its high expression strain T24 and low expression strain UM-UC-3, respectively. In T24 cells, sh-RBM11#2 and sh-RBM11#3 effectively reduced the expression of RBM11 (Figure 3A). Functional experiments showed that compared with the sh-NC group, knocking down RBM11 significantly inhibited cell migration, invasion (Figures 3B and 3C), and wound healing ability (Figures 3D and 3E), and enhanced chemotherapy sensitivity to cisplatin (Figure 3F). In addition, overexpression of RBM11 in T24 cells yielded results opposite to those mentioned above (Figure S1). We further validated in UM-UC-3 cells. We constructed an RBM11 overexpression model in UM-UC-3 cells (Figures 3G–3I). Compared with the carrier group, OE-RBM11 significantly promoted cell migration, invasion (Figures 3J and 3K), and wound healing (Figures 3L and 3M), while reducing chemotherapy sensitivity to cisplatin (Figure 3N). The above results together show that RBM11 plays a role in promoting cancer in BCa cells and affects their sensitivity to chemotherapy drugs.

### RBM11 promotes proliferation and tumor progression in BCa

**Figure 4:**
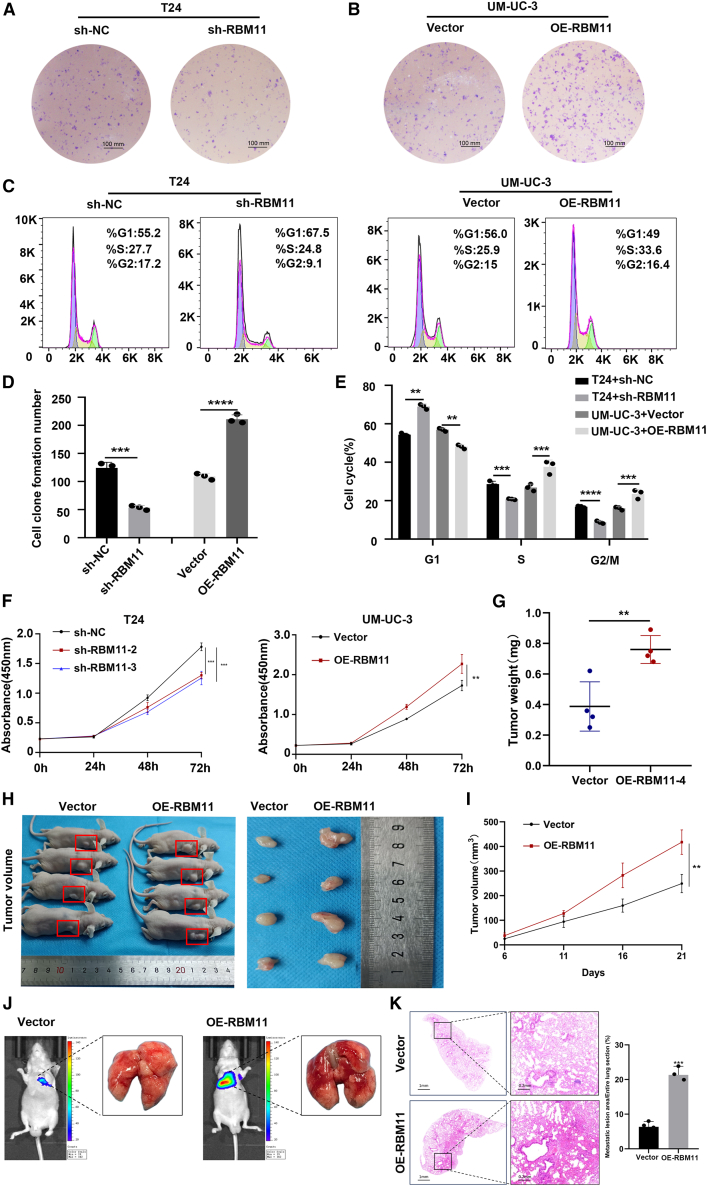
RBM11 promotes proliferation and tumor growth in BCa cells (A, B, and D) Plate colony formation assays in T24 (A) and UM-UC-3 (B) cells. *n* = 3 technical replicates from three biological replicates for each group (D). Scale bars, 100 μm. Data are presented as mean ± SD, ∗∗∗*p* < 0.001, ∗∗∗∗*p* < 0.0001 (unpaired *t* test). (C and E) Knockdown of RBM11 impeded cell cycle progression in T24 cell, whereas overexpression of RBM11 accelerated cell cycle progression in UM-UC-3 cell (*n* = 3 technical replicates from three biological replicates for each group). Data are presented as mean ± SD, ∗∗*p* < 0.01, ∗∗∗*p* < 0.001, ∗∗∗∗*p* < 0.0001 (unpaired *t* test). (F) The CCK-8 assay revealed that RBM11 knockdown inhibited cell proliferation. Overexpression of RBM11 promoted proliferation (*n* = 3 technical replicates from 3 biological replicates for each group). Data are presented as mean ± SD, ∗∗∗*p* < 0.001, ∗∗∗∗*p* < 0.0001 (two-way ANOVA). (G–I) The vivo xenograft model demonstrated that RBM11 overexpression significantly promoted tumor growth (*n* = 3 technical replicates from four biological replicates for each group). Data are presented as mean ± SD, ∗∗*p* < 0.01 (tumor weight: unpaired *t* test; tumor volume: two-way ANOVA). (J and K) The metastasis model and evaluated the number of lung metastatic foci in mice via H&E staining (*n* = 3 technical replicates from three biological replicates for each group). Data are presented as mean ± SD, ∗∗∗*p* < 0.001 (unpaired *t* test).

We evaluated the regulatory effect of RBM11 on the proliferation of BCa cells *in vitro* and *in vivo*. The colony formation assay showed that RBM11 knockdown significantly reduced the number of colonies formed by T24 cells, while RBM11 overexpression markedly increased colony formation in UM-UC-3 cells (Figures 4A, 4B, and 4D). Cell cycle analysis further revealed that RBM11 depletion induced cell cycle arrest in T24 cells, whereas RBM11 overexpression promoted cell cycle progression in UM-UC-3 cells (Figures 4C and 4E). Consistent with these findings, CCK-8 assays confirmed that RBM11 overexpression enhanced the cell proliferation rate, while RBM11 knockdown suppressed it (Figure 4F). In a xenograft tumor model, tumors formed by OE-RBM11 cells exhibited significantly larger volume and weight, as well as a higher growth rate, compared to those in the vector control group (Figures 4G–4I). We further constructed a metastasis model and evaluated the number of lung metastatic foci in mice via H&E staining. It was found that compared with the control group, the number of lung metastatic foci in mice of the RBM11-overexpressing group was significantly increased (Figures 4J and 4K). Taken together, these results demonstrate that RBM11 acts as a key regulator promoting BCa progression.

### RBM11 promotes BCa progression by activating the GNPDA1

**Figure 5:**
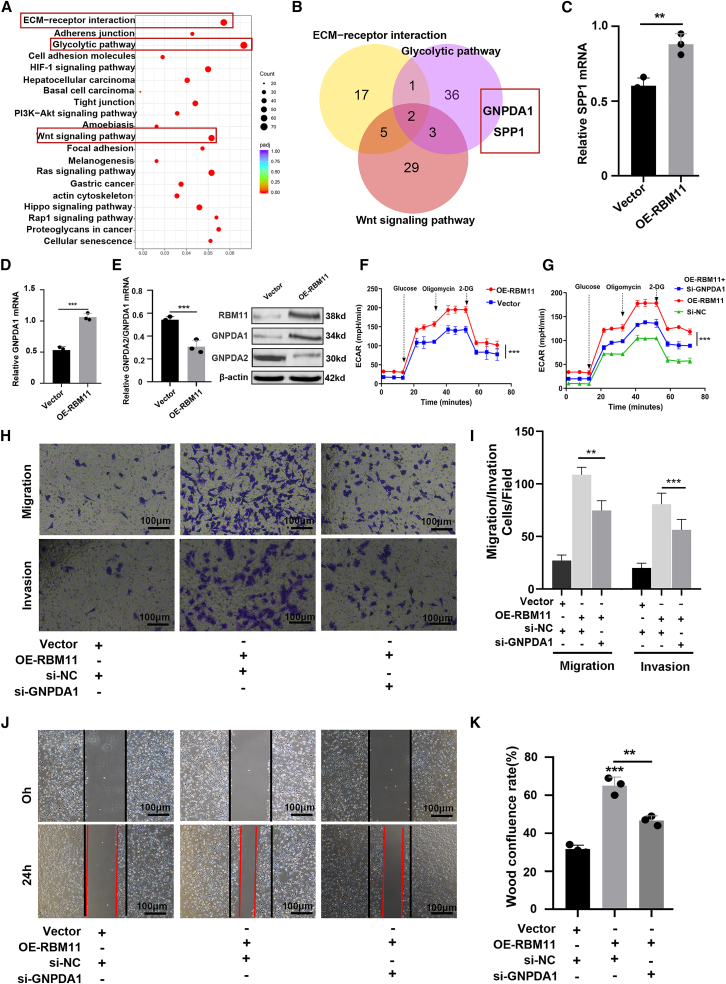
RBM11 promotes BCa progression by activating the GNPDA1 (A) RNA-seq in control and RBM11-overexpressing cells, KEGG assay revealed that DEGs significant enrichment in glycolytic pathway and extracellular matrix organization. (B) Among the coregulated genes, GNPDA1 and SPP1 showed the most pronounced upregulation. (C and D) RT-qPCR revealed that GNPDA1 was more significantly regulated by RBM11 (*n* = 3 technical replicates from three biological replicates for each group). Data are presented as mean ± SD, ∗∗*p* < 0.01, ∗∗∗*p* < 0.001 (unpaired *t* test). (E) RT-qPCR and WB revealed that OE-RBM11 significantly increased GNPDA1 while decreasing GNPDA2 (*n* = 3 technical replicates from 3 biological replicates for each group). Data are presented as mean ± SD, ∗∗*p* < 0.01 (unpaired *t* test). (F and G) Seahorse assay revealed that RBM11 overexpression promoted glycolysis (F), while GNPDA1 inhibition reduced it (G). *n* = 3 technical replicates from three biological replicates for each group. Data are presented as mean ± SD, ∗∗∗*p* < 0.001 (two-way ANOVA). (H and I) Transwell assay revealed that downregulation of GNPDA1 attenuated the invasion and migration of BCa cells. *n* = 3 technical replicates from three biological replicates for each group. Scale bars, 100 μm. Data are presented as mean ± SD, ∗∗*p* < 0.01, ∗∗∗*p* < 0.001 (one-way ANOVA). (J and K) Wound healing assay revealed that downregulation of GNPDA1 attenuated the migration of BCa cells. *n* = 3 technical replicates from three biological replicates for each group. Scale bars, 100 μm. Data are presented as mean ± SD, ∗∗*p* < 0.01, ∗∗∗*p* < 0.001 (one-way ANOVA).

In order to study how RBM11 regulates the progression of BCa, we performed RNA-seq in control and RBM11-overexpressing cells. Differential expression analysis identified significant enrichment in glycolytic pathway and extracellular matrix organization (Figure 5A). Among the coregulated genes, GNPDA1 showed the most pronounced upregulation (Figures 5B–5D). Given RBM11’s known role in RNA splicing, we examined GNPDA1 isoform expression. RBM11 overexpression significantly increased GNPDA1 while decreasing GNPDA2 (Figure 5E), indicating that RBM11 promotes a splicing shift toward the GNPDA1 isoform. We further knocked down RBM11 in T24 cells and found that the expression level of GNPDA1 was also significantly downregulated (Figure S2). Functional validation demonstrated that GNPDA1 knockdown reversed RBM11-enhanced glycolysis (Figures 5F and 5G) and attenuated RBM11-driven proliferation, migration, and invasion (Figures 5H–5K). In summary, RBM11 promotes BCa progression by regulating GNPDA alternative splicing to enhance GNPDA1 expression, thereby driving glycolytic metabolism and malignant phenotypes.

### GNPDA1 stabilizes PKM2 by inhibiting its ubiquitination and degradation in BCa

**Figure 6:**
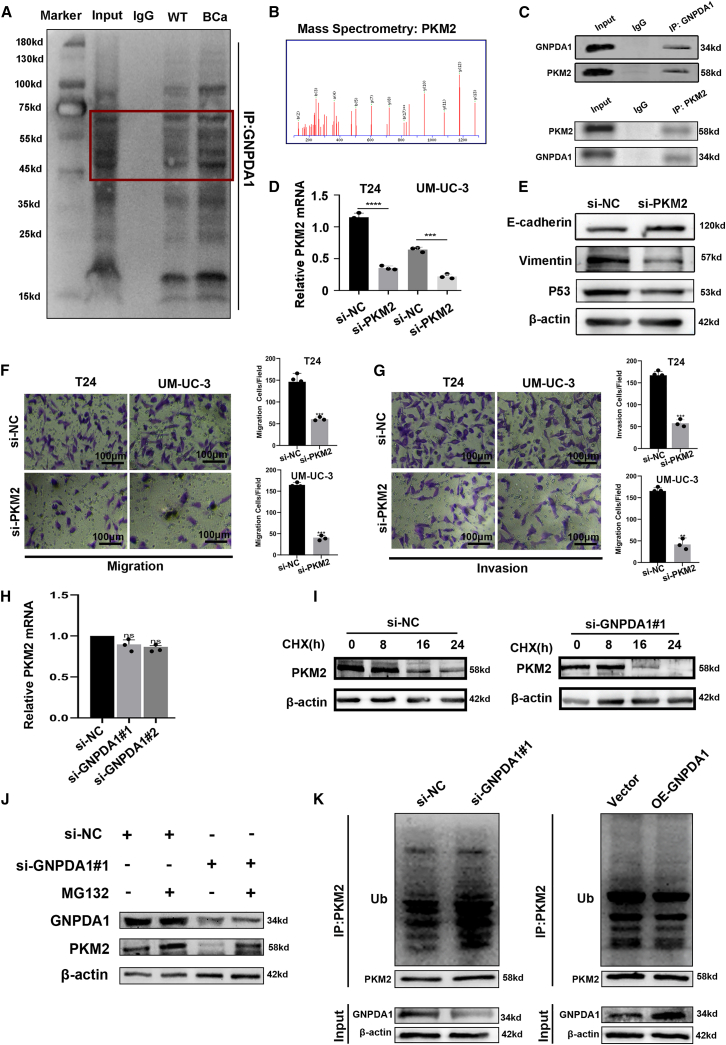
GNPDA1 stabilizes PKM2 by inhibiting its ubiquitination and degradation in BCa (A) IP was performed using anti-GNPDA1 antibody and IgG, and visualized by silver staining. (B) MS analysis showed specific binding between PKM2 and GNPDA1. (C) coIP assays demonstrated that GNPDA1 interacts with PKM2. (D) RT-qPCR detection of PKM2 knockdown efficiency. *n* = 3 technical replicates from three biological replicates for each group. Data are presented as mean ± SD, ∗∗∗*p* < 0.0001 (unpaired *t* test). (E) WB detection of inhibition of PKM2 suppresses expression of EMT-related proteins. (F and G) Transwell assay revealed that inhibition of PKM2 suppresses the invasion and migration of BCa cells. *n* = 3 technical replicates from three biological replicates for each group. Scale bars, 100 μm. Data are presented as mean ± SD, ∗∗*p* < 0.01, ∗∗∗*p* < 0.001 (unpaired *t* test). (H) RT-qPCR detection of PKM2 expression in different groups. *n* = 3 technical replicates from three biological replicates for each group. Data are presented as mean ± SD, ns stands for no statistical significance (one-way ANOVA). (I) WB detection of PKM2 stability in different groups. (J and K) GNPDA1 inhibits PKM2 ubiquitin-proteasome-mediated degradation, thereby stabilizing PKM2.

To investigate the mechanism of GNPDA1 in the malignant progression of BCa, we identified that GNPDA1 can interact specifically with PKM2 through IP-MS (Figures 6A and 6B). Co-immunoprecipitation (coIP) assays demonstrated that GNPDA1 interacts with PKM2 (Figure 6C). To further evaluate the role of PKM2 in the malignant phenotype of BCa, we used siRNA to knock down the expression of PKM2 (Figure 6D). Furthermore, we found that PKM2 knockdown led to an upregulated expression of E-cadherin, whereas the expressions of Vimentin and P53 were suppressed (Figure 6E). Additionally, PKM2 knockdown markedly inhibited the invasion and metastasis of BCa cells (Figures 6F and 6G). Notably, GNPDA1 regulation of PKM2 did not occur at the transcriptional level (Figure 6H), suggesting that GNPDA1 may modulate PKM2 through post-translational mechanisms. To test this hypothesis, we assessed protein stability using CHX chase assays. GNPDA1 knockdown markedly accelerated PKM2 degradation (Figure 6I) and enhanced its ubiquitination (Figure 6K). Conversely, treatment with the proteasome inhibitor MG132 effectively rescued PKM2 degradation induced by GNPDA1 loss (Figure 6J). Collectively, our findings demonstrate that GNPDA1 binds to PKM2 and inhibits its ubiquitin-proteasome-mediated degradation, thereby stabilizing PKM2 and promoting invasion and metastasis in BCa.

## Discussion

This study systematically elucidates the oncogenic role and molecular mechanisms of the RNA-binding protein RBM11 in BCa progression through integrated bioinformatics analysis, clinical tissue validation, and *in vitro* functional experiments. Our findings reveal that RBM11 is significantly upregulated in BCa tissues and is closely associated with poor patient prognosis. Mechanistically, RBM11 promotes GNPDA1 expression by regulating alternative splicing, thereby stabilizing PKM2 protein and promoting glycolytic metabolism, which drive BCa cell proliferation, invasion, metastasis, and chemoresistance.

EMT is a core biological process through which tumor cells acquire invasive and metastatic capabilities,^9^ and it is closely associated with the progression,^18^ chemoresistance,^19^ and poor prognosis of BCa.^20^ Our previous studies have demonstrated that inhibition of autophagy sustains the EMT process and promotes malignant progression in BCa.^21^ Strong activation of EMT signaling enhances tumor cell migration and invasion by disrupting cell polarity and intercellular junctions, reinforcing cancer stem cell properties, and remodeling the TME.^22^^,^^23^^,^^24^ Meanwhile, EMT activation can also induce chemoresistance through multiple mechanisms. Studies have shown that activated EMT signaling promotes chemotherapy resistance by modulating the DNA damage response (DDR) pathway to facilitate the repair of damaged DNA,^25^^,^^26^ while concurrently suppressing apoptosis and enhancing drug efflux capacity.^27^ Importantly, RBM11-mediated EMT activation and metabolic reprogramming may cooperatively contribute to cisplatin resistance through multiple interconnected pathways, including enhanced DNA damage repair, inhibition of apoptosis, and regulation of drug efflux transporters.^28^ In summary, EMT represents a dynamic and highly complex biological process during tumor progression, involving multiple signaling pathways, transcription factors, and epigenetic modifications. A deeper understanding of the molecular mechanisms of EMT is crucial for developing effective therapeutic strategies against tumor progression and metastasis. However, there are currently no effective clinical approaches to suppress EMT for delaying BCa progression or reversing chemotherapy resistance.

Our study demonstrates that RBM11 is significantly upregulated in BCa tissues, and its expression level is significantly correlated with tumor stage and patient prognosis, suggesting its potential role as a key oncogenic regulator in disease progression. As an RNA-binding protein, RBM11 modulates cancer cell behaviors by regulating alternative splicing, mRNA stability, and translation efficiency of target genes.^29^ Specifically, RBM11 can bind to the 3′UTR regions of EMT-related genes (E-cadherin, Vimentin, and Snail), enhancing their post-transcriptional stability and thereby promoting EMT,^30^ which in turn strengthens tumor cell migration and invasion capabilities. Furthermore, RBM11 interacts with the PP2A protein complex,^31^ inhibiting PP2A-mediated tumor-suppressive signaling and abrogating cell cycle control, ultimately leading to uncontrolled proliferation.

Notably, the oncogenic functions of RBM11 exhibit tumor type-specific characteristics: on the one hand, it can promote tumor angiogenesis by regulating the expression of angiogenic factors such as VEGF^30^; on the other hand, tumor cell apoptosis can also be inhibited by affecting the splicing patterns of apoptosis-related genes, thereby promoting tumor progression.^11^ This specificity may arise from differential RBM11 target engagement across TMEs, underscoring the complexity of RNA-binding protein regulatory networks.

Metabolic reprogramming and mRNA aberrant alternative splicing are hallmarks of cancer and are intricately linked during tumor progression.^32^^,^^33^ Enhanced glycolysis (the Warburg effect) is a central feature of metabolic rewiring, enabling cancer cells to preferentially utilize glycolysis for energy production even under aerobic conditions to support rapid proliferation.^34^^,^^35^ However, the key splicing events governing this process remain incompletely understood. Previous studies have indicated that RNA splicing can directly regulate tumor metabolism^36^; for instance, mTOR signaling influences lipid metabolism by modulating splicing of lipogenesis-related mRNAs,^37^ and switching of PKM splicing isoforms can reverse the Warburg effect.^38^ Several splicing regulators, such as SRSF1^39^ and hnRNPA1,^40^ have been reported to modulate tumor metabolic reprogramming by controlling the alternative splicing of key metabolic enzymes, thereby promoting aerobic glycolysis and tumor progression. In contrast to these previously reported splicing factors, our study identifies RBM11 as a important regulator linking alternative splicing to metabolic reprogramming in BCa. We demonstrate that RBM11 promotes glycolysis not only by shifting GNPDA pre-mRNA splicing toward the oncogenic isoform GNPDA1 but also by stabilizing PKM2 protein through inhibition of ubiquitin-proteasome-mediated degradation, revealing a dual regulatory mechanism at both the post-transcriptional and post-translational levels.

Although RBM11 has been implicated in cytoskeletal remodeling and cell cycle regulation, its role in tumor metabolic reprogramming has been unclear. Here, we further demonstrate that RBM11 governs the alternative splicing of GNPDA pre-mRNA, shifting the balance toward the oncogenic isoform GNPDA1. GNPDA1, a key regulator of glycolysis, modulates tumor cell proliferation by affecting post-translational modifications and maintaining cellular energy supply,^41^ yet its function in BCa had not been systematically investigated. We show that elevated GNPDA1 directly interacts with PKM2 and impedes its ubiquitin proteasome-mediated degradation, significantly prolonging its half-life and enhancing protein stability. This regulation augments glycolytic flux in BCa cells, providing energetic support for tumor proliferation and metastasis, thereby promoting invasive and migratory behaviors.

In recent years, emerging gene therapy strategies have shown promising potential in the treatment of BCa, particularly approaches targeting aberrant gene expression and post-transcriptional regulation. RNA interference (RNAi)-based therapies have been widely explored to selectively silence oncogenes and metabolic regulators, while antisense oligonucleotides (ASOs) have demonstrated efficacy in modulating alternative splicing events associated with tumor progression.^42^ In addition, splicing modulation strategies using small molecules or splice-switching oligonucleotides have gained increasing attention important therapeutic tools for targeting dysregulated RNA splicing in cancer.^43^ Within this therapeutic context, RBM11 represents a highly attractive candidate target. Given its central role in regulating alternative splicing and metabolic reprogramming through the GNPDA1-PKM2 axis, therapeutic inhibition of RBM11 or disruption of its splicing regulatory activity may effectively suppress tumor growth, EMT activation, and malignant progression. Targeting RBM11 using RNAi-based approaches, splicing-modulating agents, or gene-editing technologies may therefore provide innovative avenues for precision therapy in BCa.

### Limitations of the study

This study has several limitations that should be acknowledged. Although our *in vitro* and *in vivo* experiments demonstrated the oncogenic role of RBM11 in BCa progression, the subcutaneous xenograft model mainly reflects tumor growth and does not fully recapitulate the native TME, local invasion patterns, or metastatic behavior observed in patients. More physiologically relevant orthotopic BCa models will be required to further validate the role of RBM11 in tumor invasion and metastasis. In addition, the mechanistic depth of RBM11-mediated regulation of glycolysis and EMT, as well as its involvement in chemotherapy resistance, has not been fully elucidated and warrants further investigation. Moreover, effective therapeutic strategies targeting RBM11 or disrupting its downstream signaling axis are currently lacking. Future studies will therefore focus on addressing these limitations to provide effective therapeutic targets for the treatment of BCa.

## Resource availability

### Lead contact

Requests for further information and resources should be directed to and will be fulfilled by the lead contact, Weiyang He (weiyang1262020@126.com).

### Materials availability

The unique materials generated in this study are available from the lead contact upon request.

### Data and code availability

• The RNA-seq data have been submitted to the National Center for Biotechnology Information with the accession number PRJNA1426904 with full open access (https://www.ncbi.nlm.nih.gov/bioproject/PRJNA1426904).
• The mass spectrometry proteomics data have been deposited to the ProteomeXchange Consortium via the iProX partner repository^44^^,^^45^ with the dataset identifier PXD074921 (full open access, https://proteomecentral.proteomexchange.org/?pxid=PXD074921).
• This article does not report original code.
• Any additional information required to reanalyze the data reported in this article is available from the lead contact upon request.

## Acknowledgments

This study was supported by the Natural Science Foundation Project of Chongqing (CSTB2024NSCQ-MSX1163) and the 10.13039/501100001809National Natural Science Foundation of China (no. 82372881).

## Author contributions

H.T. and W.H. designed the research and revised the manuscript; H.T., T.L., and Y.S. performed the experiments and wrote the draft manuscript; J.Z., Q.D., and Q.Y. performed the experiments and analyzed the experimental results; Y.S. and W.H. engaged in reviewing and editing the article. All authors contributed to the article and approved the submitted version.

## Declaration of interests

The authors declare no competing interests.

## STAR★Methods

### Key resources table

**Table:** 

REAGENT or RESOURCE	SOURCE	IDENTIFIER
**Antibodies**		

RBM11	Abcam	Cat# ab69358, RRID:AB_2296838
GAPDH	Proteintech	Cat# 60004-1-lg, RRID:AB_2107436
β-Actin	Proteintech	Cat# 20536-1-AP, RRID:AB_10700003
E-cadherin	Abcam	Cat# ab40772, RRID:AB_11157002
Vimentin	Abcam	Cat# ab92047, RRID:AB_10562134
P53	Abcam	Cat# ab32049, RRID:AB_776982
PKM2	Abcam	Cat# ab85555, RRID:AB_10562282
Ubiquitin	Abcam	Cat# ab7780, RRID:AB_305918
GNPDA1	Proteintech	Cat# 12312-1-AP, RRID:AB_2890170

**Biological samples**		

Bladder cancer tissues and adjacent normal tissues	Department of Urology, The First Affiliated Hospital of Chongqing Medical University	–

**Chemicals, peptides, and recombinant proteins**		

Lipomaster 2000	Vazyme	Cat# TL201
Trizol reagent	Thermo Scientific	Cat# 15596018
Matrigel	Corning	Cat# 354234
4% paraformaldehyde	Biosharp	Cat# BL539A
Hematoxylin	Servicebio	Cat# G1004
Eosin	Servicebio	Cat# G1001
PureProteome Protein A/G magnetic beads	Millipore	Cat# LSKMAGAG10
PVDF membranes	Bio-Rad Laboratories	Cat# 1620177

**Critical commercial assays**		

ChamQ Universal SYBR qPCR Master Mix	Vazyme	Cat# Q711
PureBinding®RNA Immunoprecipitation Kit	GENESEED	Cat# P0102

**Deposited data**		

RNA-seq data	NCBI	SRA accession: PRJNA1240785
The mass spectrometry proteomics data	ProteomeXchange Consortium	ID: PXD074921

**Experimental models: Cell lines**		

T24	Cell Bank of Chinese Academy of Sciences, Shanghai	Cat# SCSP-536
UM-UC-3	Cell Bank of Chinese Academy of Sciences, Shanghai	Cat# SCSP-563
5637	Cell Bank of Chinese Academy of Sciences, Shanghai	Cat# SCSP-537
SV-HUC-1	Cell Bank of Chinese Academy of Sciences, Shanghai	Cat# SCSP-5382

**Experimental models: Organisms/strains**		

BALB/c nude mice	Vital River Laboratories, Beijing	–

**Oligonucleotides**		

See Tables S1 and S2	Generay	–

### Experimental model and study participant details

#### Cell culture

T24, UM-UC-3, 5637, and SV-HUC-1 human bladder cell lines were obtained from the Cell Bank of the Chinese Academy of Sciences (Shanghai, China). All cell lines were authenticated by short tandem repeat (STR) profiling and confirmed to be mycoplasma-free by a third-party service (Guangzhou Cellcook Biotech Co., Ltd.). Cells were cultured at 37 °C in a humidified incubator with 5% CO_2_ using complete medium consisting of basal medium supplemented with 10–15% fetal bovine serum (FBS) and 1% penicillin–streptomycin. SV-HUC-1 cells were maintained in F-12K medium, UM-UC-3 cells in DMEM, and T24 and 5637 cells in RPMI-1640 medium. For RBM11 knockdown, T24 cells were transfected with specific shRNAs or siRNAs targeting RBM11 using Lipofectamine 2000 according to the manufacturer’s instructions. For RBM11 overexpression, UM-UC-3 cells were infected with lentivirus encoding full-length RBM11 or empty vector at a multiplicity of infection (MOI) of 50. After 24 h, the medium was replaced, and stable cell lines were selected using puromycin (1.5 μg/mL). Knockdown and overexpression efficiencies were validated by RT-qPCR and Western blot analysis.

#### Experimental animals

All animal procedures were approved by the Institutional Animal Care and Use Committee of The First Affiliated Hospital of Chongqing Medical University (IACUC-CQMU-2022-K560) and conducted in accordance with NIH guidelines. For the xenograft model, BALB/c nude mice (4-6 weeks old, male) were subcutaneously injected with 5×10^6^ Vector or OE-RBM11 cells. Tumor volume (0.5×length×width2) was measured every 5 days, and tumors were excised and weighed after three weeks. For the metastatic model, UM-UC-3 cells in the logarithmic growth phase were harvested, washed with PBS, and resuspended at a density of 3 × 10^7^ cells/mL. Male BALB/c nude mice were randomly assigned to groups and intravenously injected with 100 μL of cell suspension via the lateral tail vein. For bioluminescence imaging, mice were anesthetized with isoflurane and intraperitoneally injected with D-luciferin potassium salt, followed by imaging after 5 min using an *in vivo* imaging system. Lung metastasis was monitored at 3 weeks post-injection. After imaging, mice were sacrificed, and lungs were collected, fixed in 4% paraformaldehyde, embedded in paraffin, sectioned, and stained with H&E for metastatic lesion assessment.

#### Ethical approval

The bladder cancer tissues and normal adjacent tissues for analysis were sourced from patients undergoing surgery from 2022 to 2025 at The First Affiliated Hospital of Chongqing Medical University. In this investigation, the samples utilized were the residual tissues following pathological examination. Throughout the study and upon the release of the findings, the personal information of patients was strictly safeguarded and not disclosed. Human bladder cancer samples were collected and investigated in line with the Declaration of Helsinki. Ethical approval was obtained from the First Affiliated Hospital of Chongqing Medical University ethics committees prior to the commencement of the study (No. 2022-86). Written informed consent was obtained from all participants. A total of 20 pairs of human tissue samples (numbered 1–20), comprising 20 tumor tissues (T1–T20) and 20 matched adjacent non-cancerous tissues (N1–N20), were enrolled in this study. All samples were subjected to detection of RBM11 transcription level via qRT-PCR. For immunohistochemistry (IHC) analysis, simple randomization was employed for sample allocation: 6 adjacent non-cancerous tissues (N) were randomly assigned to the normal group; 6 tumor tissues at stage I–II were randomly assigned to the stage I–II group; and 6 tumor tissues at stage III–IV were randomly assigned to the stage III–IV group.

### Method details

#### Bioinformatics analyses

Transcriptome and clinical data from bladder cancer samples were obtained from TCGA (https://portal.gdc.cancer.gov/). Using the “Hallmark Epithelial-Mesenchymal Transition” gene set (https://www.gsea-msigdb.org/), 405 patients were stratified into EMT-up and EMT-down groups based on the expression of 200 EMT-associated genes. Kaplan-Meier analysis assessed survival differences. Differential expression analysis between EMT-up/down groups and tumor/normal tissues identified DEGs using the “limma” R package. EMT-associated lncRNAs were screened via Pearson correlation with the EMT gene set. Overlapping genes between DEGs and EMT-associated genes were identified using Biovenn.

#### Immunohistochemical staining

Paraffin-embedded tissue sections were baked at 60°C for 30 min, followed by deparaffinization in xylene and rehydration through a graded ethanol series. Sections were rinsed with distilled water and incubated with 3% H_2_O_2_ at room temperature for 5-10 min to block endogenous peroxidase activity. Antigen retrieval was performed in citrate buffer using a pressure cooker for 15 min. After cooling, sections were washed with PBS and blocked with 10% goat serum for 30 min at room temperature. Sections were then incubated with primary antibodies at 4°C overnight. After PBS washes, sections were incubated with biotinylated secondary antibodies for 1 h at room temperature, followed by horseradish peroxidase–conjugated streptavidin. Signal detection was performed using DAB substrate, and nuclei were counterstained with hematoxylin. Finally, sections were dehydrated, cleared, and mounted for microscopic analysis.

#### Quantitative real-time PCR (RT-qPCR)

Total RNA was extracted using TRIzol reagent according to the manufacturer’s instructions. One microgram of total RNA was reverse-transcribed into cDNA using a reverse transcription kit following the manufacturer’s protocol. Quantitative PCR was performed using ChamQ Universal SYBR qPCR Master Mix on a real-time PCR system. Each reaction was carried out in a 20 μL volume, and amplification was performed under the following conditions: initial denaturation at 95°C for 30 s, followed by 40 cycles of denaturation at 95°C for 10 s and annealing/extension at 60°C for 30 s. GAPDH was used as the internal control for normalization. Relative gene expression levels were calculated using the 2^-^ΔΔCt method. All reactions were performed in triplicate, and experiments were repeated at least three times independently. Primer sequences are listed in Table S2.

#### Western blot analysis

Total protein was extracted using RIPA lysis buffer supplemented with PMSF and quantified using a BCA protein assay kit. Equal amounts of protein (20–30 μg) were separated by SDS-PAGE and transferred onto PVDF membranes. Membranes were blocked with 5% non-fat milk for 1 h at room temperature and incubated with primary antibodies overnight at 4°C. After incubation with HRP-conjugated secondary antibodies for 1 h at room temperature, protein bands were visualized using enhanced chemiluminescence (ECL). Antibody information and dilutions are listed in the key resources table. Band intensities were quantified using ImageJ software. Target protein expression levels were normalized to the corresponding internal control and presented as relative values.

#### Transwell experiments

For migration assays, 5 × 10^4^ cells suspended in 200 μL serum-free medium were seeded into the upper chamber of Transwell inserts (8-μm pore size), with 600 μL complete medium containing 10% FBS added to the lower chamber as a chemoattractant. For invasion assays, the upper chamber was pre-coated with Matrigel (Corning, diluted 1:8 in serum-free medium) and allowed to solidify at 37°C for 1 h before cell seeding. After incubation at 37°C for 12 h, non-migrated cells on the upper surface were removed, and cells on the underside of the membrane were fixed with 4% paraformaldehyde, stained with 0.1% crystal violet, and imaged under a light microscope. The number of migrated or invaded cells was quantified using ImageJ software by counting cells in five randomly selected fields per membrane.

#### Wound healing assays

Cells were seeded into 6-well plates and cultured until reaching approximately 90-100% confluence. A linear wound was created by scratching the cell monolayer using a sterile 200 μL pipette tip. Detached cells were gently removed by washing twice with phosphate-buffered saline (PBS). Cells were subsequently cultured in serum-free medium to minimize the effect of cell proliferation. Images of the wound area were captured immediately after scratching (0 h) and at 24 h using an inverted light microscope. The wound width was measured using ImageJ software, and wound closure was calculated as the percentage of the initial wound area.

#### CCK-8 assay

Cell viability was assessed using a Cell Counting Kit-8 (CCK-8) assay according to the manufacturer’s instructions. Cells were seeded into 96-well plates at a density of 2 × 10^3^ cells per well in 100 μL complete medium and allowed to adhere overnight. Cells were then treated with cisplatin at the indicated concentrations (0-20 μM) for 48h. At each indicated time point, 10 μL of CCK-8 solution was added to each well and incubated at 37°C for 2 h. The absorbance at 450 nm was measured using a microplate reader. Cell viability was calculated as a percentage relative to the untreated control group.

#### Plate colony formation assays

Transfected cells were seeded into 6-well plates at a density of 500 cells per well and cultured for 7-10 days until visible colonies formed. Colonies were fixed with 4% paraformaldehyde, stained with 0.1% crystal violet, and counted manually.

#### Hematoxylin and eosin (H&E) staining

Paraffin-embedded tissue sections (3-5 μm) were prepared, baked at 60°C for 30 min, deparaffinized in xylene, and rehydrated through a graded ethanol series. Sections were stained with hematoxylin, differentiated with acid alcohol, and counterstained with eosin. After dehydration and clearing, sections were mounted with neutral resin and examined under a light microscope.

#### IP and mass spectrometry sequencing

Cells were lysed in IP lysis buffer containing protease inhibitors. Equal amounts of protein lysates (500 μg) were incubated with 2 μg of anti-GNPDA1 antibody or IgG control overnight at 4 °C with gentle rotation. Immune complexes were captured using Protein A/G magnetic beads for 2 h, washed extensively, and eluted with SDS loading buffer. Eluted proteins were separated by SDS-PAGE and visualized by Coomassie blue staining. Entire gel lanes were excised, subjected to in-gel tryptic digestion, and analyzed by LC-MS/MS. Mass spectrometry analysis and protein identification were performed by GenePharma (Shanghai, China). The mass spectrometry proteomics data have been deposited to the ProteomeXchange Consortium via the iProX partner repository with the dataset identifier PXD074921. The analytical data were compared against the human protein database to accomplish protein identification and qualitative analysis.

#### Ubiquitination test

For ubiquitination analysis, cells were treated with the proteasome inhibitor MG132 (10 μM) for 6 h prior to harvest. Cell lysates were immunoprecipitated with anti-PKM2 antibody overnight at 4 °C, followed by incubation with Protein A/G magnetic beads. Immunoprecipitated complexes were subjected to Western blot analysis using anti-ubiquitin and anti-PKM2 antibodies.

#### Cell culture and cycloheximide (CHX) experiments

To assess protein stability, cells were treated with cycloheximide (CHX; 30 μM) to inhibit *de novo* protein synthesis. Cells were harvested at indicated time points (0, 8, 16, and 24 h), and total protein was extracted for Western blot analysis. PKM2 protein levels were quantified and normalized to loading controls to determine degradation kinetics.

#### Seahorse analysis

Extracellular acidification rate (ECAR) was measured using a Seahorse XF Extracellular Flux Analyzer. Cells were seeded into Seahorse XF microplates at 2 × 10^4^ cells per well and incubated overnight. Prior to measurement, culture medium was replaced with Seahorse assay medium, and cells were equilibrated at 37 °C in a non-CO_2_ incubator for 1 h. For glycolysis stress tests, glucose, oligomycin, and 2-deoxy-D-glucose (2-DG) were sequentially injected according to the manufacturer’s protocol. ECAR values were normalized to cell number or total protein content.

#### RNA-seq

Vector and OE-RBM11 cells were seeded into 6 cm culture dishes and incubated for 24 hours to allow sufficient cell attachment. Total RNA was then extracted from each sample using RNA extraction reagent according to the manufacturer’s instructions. RNA quality and integrity were verified before library construction. RNA-seq libraries were prepared and sequenced by BGI Genomics (Shenzhen, China). Each group included three independent biological replicates. Sequencing was performed on an Illumina platform, generating paired-end reads. Raw sequencing reads were processed to remove low-quality reads and adapters. Clean reads were aligned to the human reference genome (GRCh38), and gene expression levels were quantified. Differentially expressed genes (DEGs) were identified using standard bioinformatics pipelines, with thresholds of fold change ≥ 2.0 and adjusted p value < 0.05. Functional enrichment analyses were performed using GO and KEGG analysis.

### Quantification and statistical analysis

All experiments were performed with at least three independent biological replicates. Data are presented as means ± standard deviation (SD). Statistical comparisons between two groups were determined by Student’s t-test, whereas multiple group comparisons were analyzed by one-way ANOVA. Two-way ANOVA was used for comparisons involving multiple groups and time points. All statistical computations were conducted using GraphPad Prism 10, with p < 0.05 considered statistically significant. Significant differences throughout all the figures are represented by ∗p < 0.05, ∗∗p < 0.01, ∗∗∗p < 0.001, ∗∗∗∗p < 0.0001.
