# Supplementary material for: RBM11 drives malignant progression of bladder cancer by regulating GNPDA1-PKM2 axis
Source: iScience. 2026 Mar 17;29(4):115402. doi: 10.1016/j.isci.2026.115402 (PMC13068525; doi:10.1016/j.isci.2026.115402)
Supplement: Document S1. Figures S1–S3 and Tables S1 and S2 [file mmc1.pdf]

iScience, Volume *29*

Supplemental information

RBM11 drives malignant progression of bladder cancer by regulating GNPDA1-PKM2 axis

Hang Tong, Tinghao Li, Junlong Zhu, Qian Dou, Qiong Yu, Yan Sun, and Weiyang He

Supplemental information（Figures S1-S3 and Tables S1-S2）

RBM11 drives malignant progression of bladder cancer by regulating GNPDA1/PKM2 axis

Hang Tong, Tinghao Li, Junlong Zhu, Qian Dou, Qiong Yu, Yan Sun and Weiyang He

Figure S1

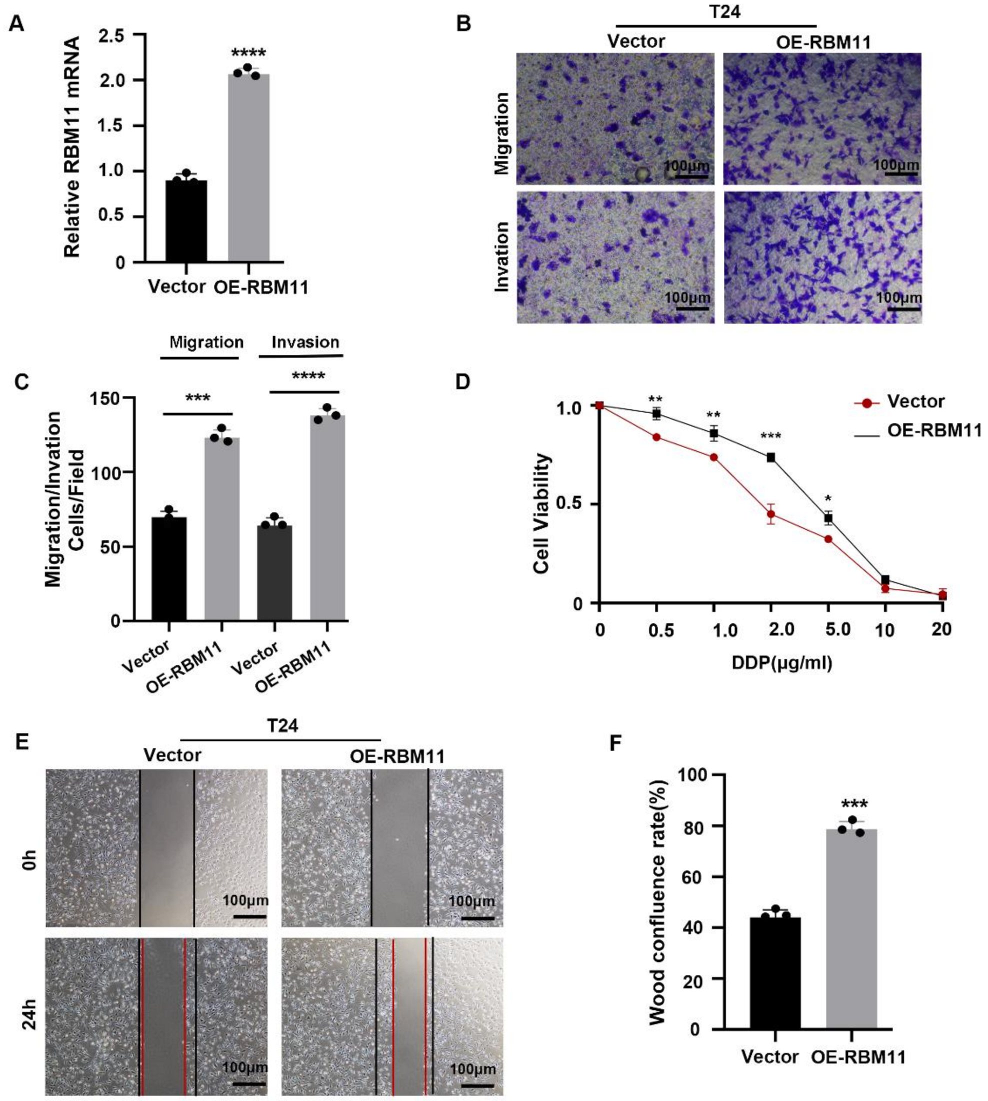

Figure S1 Overexpression of RBM11 further promotes the malignant progression of BCa.

(A) RT-qPCR detection efficiency of OE-RBM11 in T24 (n = 3 technical replicates from 3 biological replicates for each group). Data are presented as mean ± SD, ****$p < 0.0001$ (Unpaired t test).

(B and C) Transwell assays demonstrated that RBM11 overexpression enhances T24 cell migration and invasion (n = 3 technical replicates from 3 biological replicates for each group). Scale bars: panel = 100 μm. Data are presented as mean ± SD, ***$p < 0.001$, ****$p < 0.0001$ (Unpaired t test).

(D) CCK8 assays demonstrated that RBM11 overexpression inhibits T24 cell chemotherapy sensitivity (n = 3 technical replicates from 3 biological replicates for each group). Data are presented as mean ± SD, *$p < 0.05$, **$p < 0.01$, ***$p < 0.001$ (Unpaired t test).

(E and F) Wound healing assay demonstrated that RBM11 overexpression enhances T24 cell migration (n = 3 technical replicates from 3 biological replicates for each group). Scale bars: panel = 100 μm. Data are presented as mean ± SD, ***$p < 0.001$ (Unpaired t test).

Figure S2

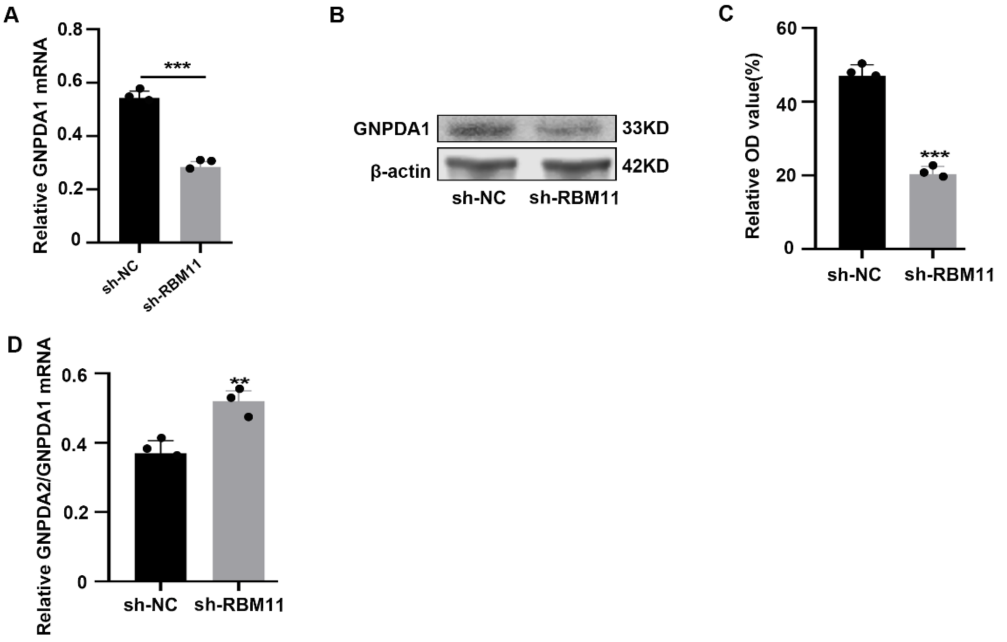

Figure S2 Knockdown of RBM11 inhibited the expression of GNPDA1.

(A) RT-qPCR revealed that GNPDA1 expression is downregulated after RBM11 knockdown (n = 3 technical replicates from 3 biological replicates for each group). Data are presented as mean ± SD, ***p < 0.001 (Unpaired t test).

(B and C) WB revealed that GNPDA1 expression is downregulated after RBM11 knockdown. (n = 3 technical replicates from 3 biological replicates for each group). Data are presented as mean ± SD, ***p < 0.001 (Unpaired t test).

(D) RT-qPCR revealed that sh-RBM11 significantly increased GNPDA2 while decreasing GNPDA1 (n = 3 technical replicates from 3 biological replicates for each group). Data are presented as mean ± SD, **p < 0.01 (Unpaired t test).

.

Figure S3

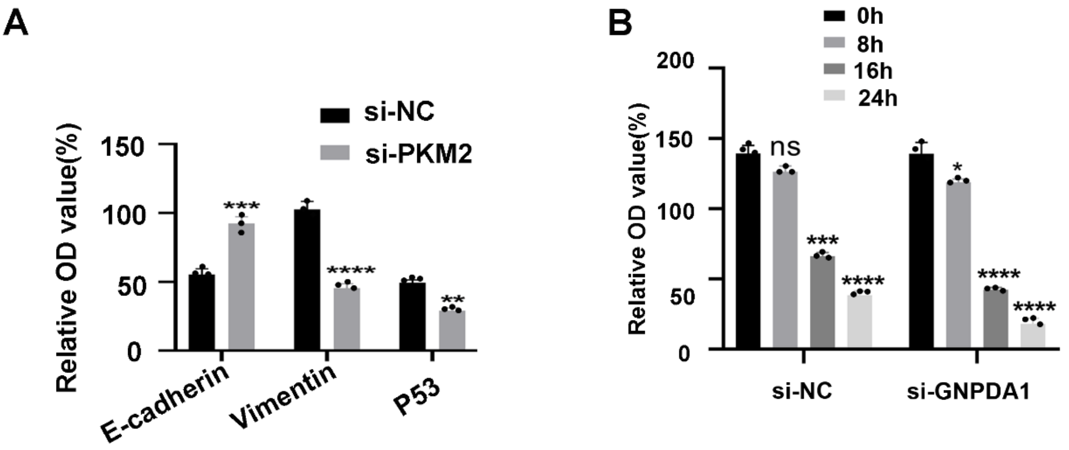

Figure S3 Supplementary Statistics Figure of Figure 6.

(A) Supplementary Statistics Figure of Figure 6E. n = 3 technical replicates from 3 biological replicates for each group. Data are presented as mean ± SD, **p < 0.01, ***p < 0.001, ***p < 0.0001 (Unpaired t test).

(B) Supplementary Statistics Figure of Figure 6I. n = 3 technical replicates from 3 biological replicates for each group. Data are presented as mean ± SD, *p < 0.05, ***p < 0.001, ***p < 0.0001 (Unpaired t test).

Table S1

Table S1. The Lentiviral and siRNA Sequence

**Gene**	**Sequence (5'-3')**
shRBM11-1	GCTGAACTTAGACCTGAAA
shRBM11-2	TTTCAGGTCTAAGTTCAGC
shRBM11-3	GCTGGAAGATTACGACAAA
shRBM11-NC	CAACAAGATGAAGAGCACCAA
siGNPDA1-1	GGAUCAAGUUCUACAACUATT
siGNPDA1-2	CCAGCAAGAUGAUGAUCAUTT
siGNPDA1-NC	UUCUCCGAACGUGUCACGUTT
siPKM2	GGACGAUAUCAAGAAUGUATT
siPKM2-NC	UUCUCCGAACGUGUCACGUTT

Table S2

Table 2. The PCR Primer Sequence

**Gene**	**Target sequence (5'-3')**
RBM11	GAGGAGCCGGAGCCAGAA
	CTCCTCCACCTTCACCATCG
GNPDA1	CCTGGATTGAGATGAAGATGAAG
	GGTCCTTGTAGATGCCATTGG
PKM2	CCAGAGGAGCCAGAATGG
	CACAGGCATTCCGGGTT
SPP1	GATGATGATGACGACGACG
	GTCCTTGGGGTCTAGGACTG
GNPDA2	CAGCAAGATCATGATCAGCTG
	GTTCTTGATGCCATTGGTGTAG
